# A Case Report of Necrotizing Pneumonia Due to Burkholderia cepacia Syndrome

**DOI:** 10.7759/cureus.52955

**Published:** 2024-01-25

**Authors:** Antoine S Hedary, Nikki L DeBord, Diahann Marshall

**Affiliations:** 1 Family Medicine, Louisiana State University Health Sciences Center, Alexandria, USA

**Keywords:** burkholderia cepacia complex, severe sepsis, antibiotic-resistant bacteria, immunocompetent patients, necrotizing peumonia

## Abstract

*Burkholderia cepacia* ​​​​​​(*B. cepacia*) complex is a highly resistant gram-negative pathogen known to cause lung infection in cystic fibrosis, chronic granulomatous disease, and immunocompromised patients. However, it may rarely infect immunocompetent patients as well. Here, we present the case of a 30-year-old male patient who was treated for *B. cepacia* pneumonia in the hospital, discharged with oral antibiotics, and returned two months later with recurring *B. cepacia* pneumonia and bacteremia. The patient rapidly declined over the next 24 hours and expired in the intensive care unit. This case is significant as it is one of very few published cases of cepacia syndrome in a patient with no evidence of immunodeficiency. In conclusion, cases of *B. cepacia* pneumonia must be monitored vigilantly for progression to cepacia syndrome, even in immunocompetent patients. Additional studies regarding optimized antibiotic regimens and effective treatment modalities for *B. cepacia* infection are warranted.

## Introduction

*Burkholderia cepacia* (*B. cepacia*) complex is a class of gram-negative bacteria found most often in soil and water [1]. They are a group of catalase-positive, obligate anaerobes, which are usually flagellated and motile, except for *B. mallei* [1-2]. Originally classified as a Pseudomonas species variant at its discovery by plant biologist Walter Burkholder in the 1950s, it was reclassified into its own genus in the early 1990s as a result of DNA hybridization and RNA sequence alignments [1]. Many species are ecologically relevant as nitrogen-fixing bacteria [1]. *B. cepacia* complex is conventionally classified into genetically differentiable subtypes with little phenotypic variation, formerly known as genomovars; these genomovars are known for being highly adaptable to many environments [1-3]. *B. cepacia* complex species are complex and exceedingly mutable, making them prone to antimicrobial resistance and presenting significant treatment challenges [1-4].

In medical settings, infection with Burkholderia species is most often seen in the immunocompromised, such as cystic fibrosis patients, chronic granulomatous disease patients, transplant recipients, and those who are elderly or infants. Cystic fibrosis is an inherited obstructive lung pathology that results in a susceptibility to certain pathogens, including Burkholderiaspecies. Patients on mechanical ventilation are also vulnerable, and *B. cepacia* complex infection can rarely be found in patients exposed to contaminated water sources or drowning victims [1-4]. About 10% of patients infected with Burkholderia progress to a state of rapid respiratory and systemic decline known as cepacia syndrome, marked by bacteremia, acute respiratory distress syndrome, and necrotizing pneumonia. Cepacia syndrome carries a mortality rate of 75% [5].

The metabolic and genotypic diversity of *B. cepacia* complex presents a diagnostic challenge. Burkholderia species are isolated from sputum culture samples using a combination of *B. cepacia*-specific agar and Oxidative-Fermentation Polymyxin-Bacitracin Lactulose, with a sensitivity of 96 and 100%, respectively. Definitive identification can be accomplished by nucleic acid amplification of 16s rRNA sequences, with a recombinase-aided amplification yielding a specificity of 98% [6-7].

Antibiotics effective against *B. cepacia* include cephalosporins, carbapenems, sulfamethoxazole/trimethoprim, chloramphenicol, and tetracyclines, the most active being minocycline (38%), meropenem (26%), and ceftazidime (23%) [2]. Notably, in one pediatric case study from 2019, a 17-year-old chronic granulomatous disease patient was successfully treated for cepacia syndrome with a combination of inhaled tobramycin and eight weeks of ceftazidime, minocycline, and Bactrim, along with extracorporeal membrane oxygenation and steroids [5-7].

## Case presentation

**Figure 1:**
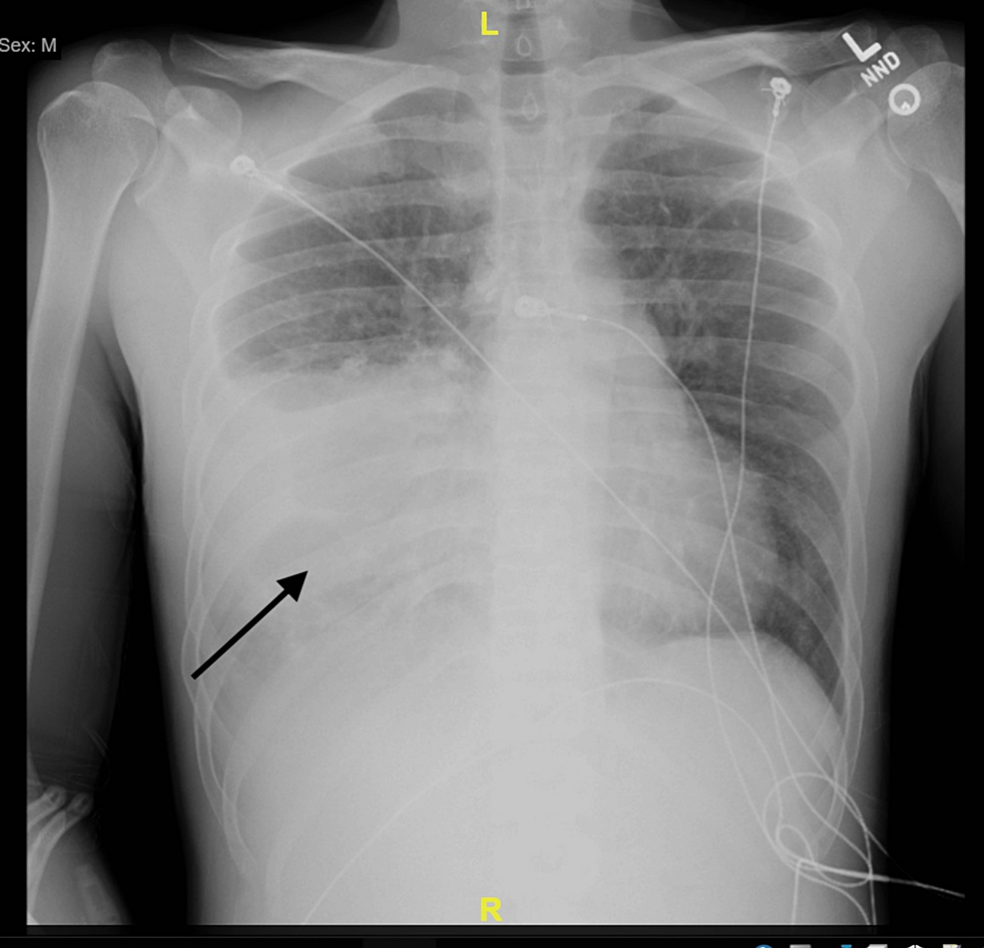
Chest radiography with right lower lobe infiltrates

**Figure 2:**
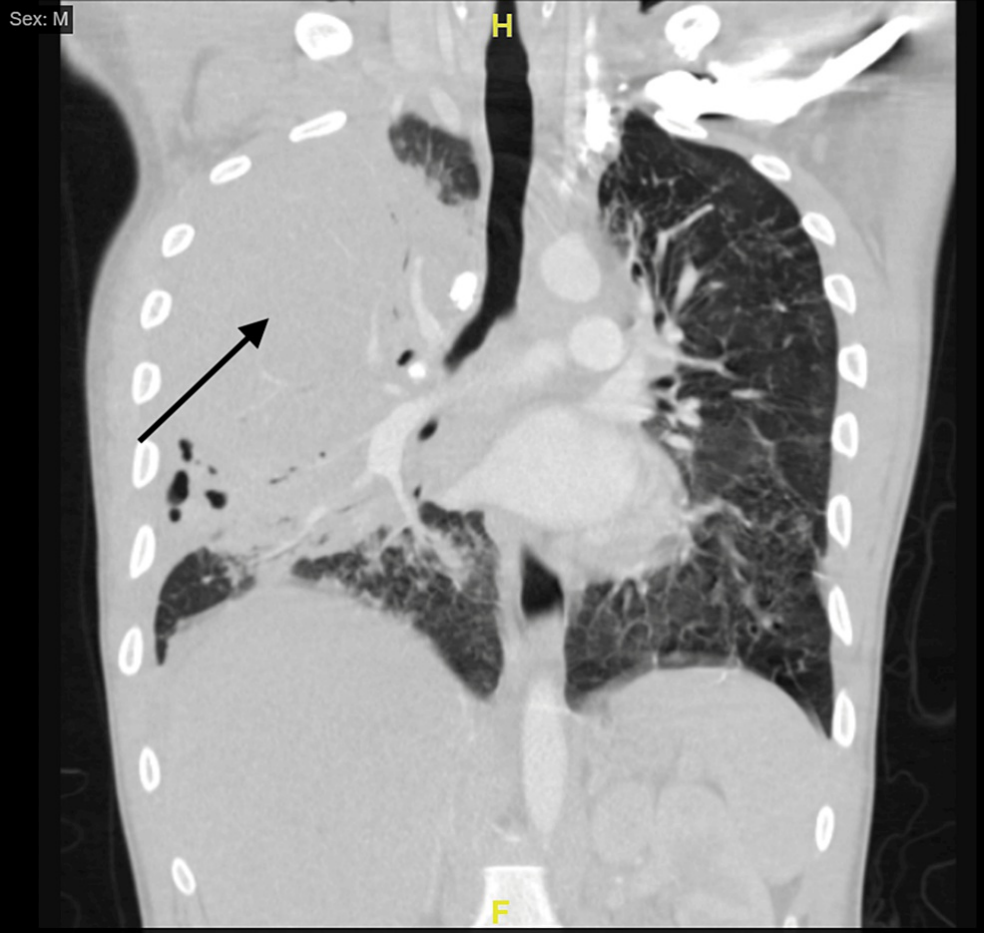
Computed tomography scan showing right-sided infiltrate with fibrosis and chronic granulomatous calcifications

A 30-year-old male patient with no medical history except smoking presented to the emergency department with nausea, dyspnea, and malaise for the past seven days. On presentation, the patient was hypoxic, tachycardic, and febrile to 103 degrees Fahrenheit. Chest radiography at the time showed large right lower lobe opacities (Figure 1). Labs showed hyponatremia with a sodium of 123 mmol/L (normal 135-148 mmol/L), hypokalemia of 2.9 mmol/L (normal 3.3-5.1 mmol/L), leukocytosis of 14.0 k/mm^3 ^(normal 5.0-10.0 k/mm^3^), and borderline elevated lactic acid of 2.6 mmol/L (normal 0.7-2.5 mmol/L). An electrocardiogram was concerning for S-T elevation, and cardiology was consulted. The troponin measured was negative at <0.01 ng/mL (normal 0.0-0.1 ng/mL), and cardiology suspected pericarditis after a repeat electrocardiogram. Computed tomography angiography was negative for any pulmonary embolism; however, it did show a suspicious mass, infiltrates, fibrosis, and chronic granulomatous calcifications (Figure 2). The patient's laboratory data for this admission is summarized in Table 1. Hepatitis, influenza A/B, COVID-19, and human immunodeficiency virus testing yielded negative results. A urinalysis was concerning for kidney injury but negative for infection.

**Table 1:** Laboratory data from the first admission

Laboratory Data From the First Admission	Lab Values	Reference Ranges
Sodium	123 mmol/L	135-148 mmol/L
Potassium	2.9 mmol/L	3.3-5.1 mmol/L
Leukocytes	14.0 k/mm^3^	5.0-10.0 k/mm^3^
Lactic Acid	2.6 mmol/L	0.7-2.5 mmol/L
Troponin	<0.01 ng/mL	0.0-0.1 ng/mL

The patient responded to a fluid bolus and was admitted to the floor on four liters of nasal cannula oxygen with intravenous ceftriaxone and azithromycin for broad respiratory coverage. Nephrology was consulted to manage hyponatremia, and hematology/oncology was consulted due to suspicion of malignancy. On hospital day one, transthoracic echocardiography demonstrated an ejection fraction of 45 to 50%, mild ventricular dilation, and mild diffuse hypokinesis. Motrin and colchicine were given for pericarditis. On day two, the patient reported improvement, and medications for pericarditis were discontinued. On day three, the patient remained stable, and hematology/oncology recommended bronchoscopy for bronchoalveolar lavage with biopsy to rule out malignancy. On day four, blood cultures drawn at admission grew *B. cepacia* sensitive to levofloxacin, ceftazidime, meropenem, and trimethoprim-sulfamethoxazole. The patient's antibiotics were then adjusted to intravenous meropenem, one gram every eight hours, and infectious disease was consulted. The infectious disease consultant noted a history of *Chromobacterium violaceum* infection at age 11 in this patient, suspecting undiagnosed chronic granulomatous disease. A bronchoscopy with bronchoalveolar lavage was done, and samples were sent to pathology. The patient continued to improve on hospital day five with intravenous meropenem. On day six, bronchoalveolar lavage and biopsy returned a negative result for malignant cells. However, they showed evidence of acute and chronic inflammation with copious amorphous granular debris, indicating a necrotic process. The patient's condition progressively improved, and he was discharged on day seven with a two-week course of trimethoprim-sulfamethoxazole to follow up with infectious disease for screening and additional tests. 

Results of the patient's fungal studies and follow-up screening for chronic granulomatous disease, cystic fibrosis, and alpha-1 antitrypsin were negative, along with normal IgM and IgG levels. The patient followed up appropriately with infectious disease and was presumably cleared of infection.

**Figure 3:**
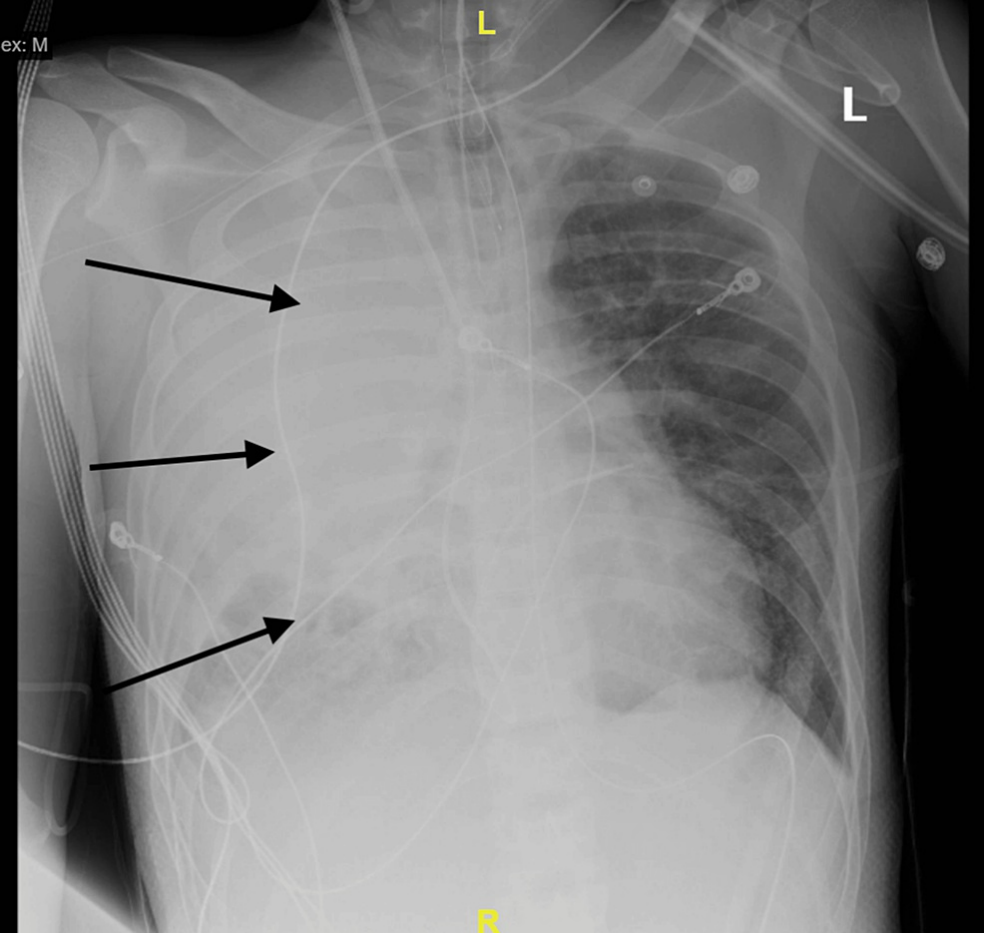
Chest radiography showing worsening right-sided infiltrates

The patient returned to the emergency department seven weeks later with similar complaints of shortness of breath, malaise, and fever for the past five days. Chest radiography showed worsened right lung consolidation when compared to the previous (Figure 3). Labs showed hyponatremia at 124 mmol/L (normal 135-148 mmol/L), hypokalemia at 3.2 mmol/L (3.3-5.1 mmol/L), elevated anion gap of 19 (normal 0-15), blood urea nitrogen of 45 mg/dL (normal 6-19 mg/dL) and creatinine of 1.77 mg/dL (normal 0.70-1.30 mg/dL), aspartate transaminase of 518 units/L (normal 0-37 units/L) and alanine transaminase of 60 units/L (normal 0-40 units/L), and lactic acid of 4.6 mmol/L (normal 0.7-2.5 mmol/L). An arterial blood gas revealed respiratory alkalosis with a pH of 7.491 (normal 7.350-7.450). The patient's laboratory data for this admission is summarized in Table 2. 

**Table 2:** Laboratory data from the second admission

Laboratory Data From the Second Admission	Lab Values	Reference Ranges
Sodium	124 mmol/L	135-148 mmol/L
Potassium	3.2 mmol/L	3.3-5.1 mmol/L
Anion Gap	19	0-15
Blood Urea Nitrogen (BUN)	45 mg/dL	6-19 mg/dL
Creatinine	1.77 mg/dL	0.70-1.30 mg/dL
Aspartate Transaminase (AST)	518 units/L	0-37 units/L
Alanine Transaminase (ALT)	60 units/L	0-40 units/L
Lactic Acid	4.6 mmol/L	0.7-2.5 mmol/L
ABG pH	7.491	7.350-7.450

The patient was bolused with lactated Ringer's and was given intravenous ceftriaxone, azithromycin, and meropenem. The hospitalist team subsequently admitted the patient for respiratory failure and severe sepsis due to pneumonia, transaminitis, acute kidney injury, and alcohol withdrawal. An infectious disease physician was consulted again when this patient was readmitted and recommended an immunology consult with CD4 and CD8 flow cytometry, testing for sickle cell disease, and a pulmonology consult.

The patient's condition remained unchanged until the evening of day one when a code blue was activated for cardiac arrest. The patient was given vasopressors, was intubated, and was placed on mechanical ventilation for respiratory support. In septic shock, the patient subsequently sustained multiple additional pulseless electrical activity events before expiring three hours later due to cardiac arrest. A review of diagnostic studies shows that the patient had likely progressed to acute respiratory distress syndrome (ARDS), with rapidly progressing opacities on chest radiography and a PaO2/FiO2 ratio of less than 100 (PaO2/FiO2 ratio less than 100 suggestive of severe ARDS.) Blood culture results returned several days post-mortem showing *B. cepacia* bacteremia, with results of the bronchoalveolar lavage showing evidence of lung necrosis. This patient's rapid decline can best be attributed to cepacia syndrome. The chronology of the patient's hospital courses is described in Table 3.

**Table 3:** Chronology of the patient's hospital admissions

	First Admission
Day 1	The patient was admitted for sepsis secondary to pneumonia with respiratory insufficiency. Pericarditis and malignancy were included in the differential, and specialists were consulted.
Day 2	The patient reported improvement on intravenous antibiotics, including ceftriaxone and azithromycin. Motrin and colchicine were discontinued as pericarditis was ruled out.
Day 3	Hematology and oncology were consulted, and bronchoscopy and bronchiolar lavage with biopsy were recommended to investigate potential malignancy. The patient remained stable.
Day 4	Blood cultures grew *Burkholderia cepacia*, and antibiotics were adjusted to intravenous meropenem. The infectious disease consultant suggested the likelihood of chronic granulomatous disease.
Day 5	The patient continued to improve on intravenous meropenem. Bronchoscopy with bronchiolar lavage and biopsy was completed, and the specimen was sent for pathology evaluation.
Day 6	Bronchiolar lavage and biopsy resulted negative for malignancy. Results did show evidence of acute and chronic inflammation with copious amorphous granular debris, indicating a necrotic process.
Day 7	The patient continued to improve, and he was discharged with a two-week course of trimethoprim-sulfamethoxazole. Follow-up with an infectious disease specialist was scheduled.
	Second Admission
Day 1	The patient was admitted for severe sepsis secondary to pneumonia with multiple-organ failure, including respiratory, renal, and hepatic systems. His condition progressed to shock and acute respiratory distress syndrome (ARDS), requiring pressors and mechanical ventilation. The patient expired following several attempts at resuscitation for cardiac arrest.

## Discussion

Cepacia syndrome is an infrequent occurrence in non-cystic fibrosis patients. Of the few cases published, most have resulted in a rapid decline and death, as in this patient [8]. Cepacia syndrome is defined by acute respiratory distress, necrotizing pneumonia, and bacteremia [7-9]. In a case study of three patients deceased from *B. cepacia* complex pneumonia, pathology studies from open lung biopsy revealed necrotizing granulomatous inflammation in all three cases, with stellate granulomas isolated from mediastinal lymph nodes in one patient. Confluent microabscesses were also seen on biopsy, similar to *Burkholderia pseudomallei* infection or melioidosis. *B. cepacia* and *B. pseudomallei* are both known to cause microabscess formation and necrotizing granulomatous inflammation with dissemination to the lymph nodes [9-10]. This similarity in pathology and disease course is highly significant for our case, where a patient with *B. cepacia* pneumonia, necrotizing granulomatous inflammation, acute respiratory distress, and sepsis rapidly declined within 48 hours of hospitalization. These findings shed some light on the causes of this patient's rapid acute decline, but the source of this patient's susceptibility to *B. cepacia *complex infection remains unclear. Radiologic and immunologic studies did not point to a clear cause. Contrast-enhanced computed tomography of the chest demonstrated left-sided fibrosis and granulomatous calcification. Mediastinal lymphadenopathy seen on computed tomography resembling malignancy or granulomatous inflammation pointed to fibrotic or granulomatous lung disease. Chest radiography done throughout the hospital stay showed diffuse opacification of the right lung and lower left lung fields; fibrosis of the left upper lung fields may explain why chest radiography did not show the bilateral diffuse whiteout seen in acute respiratory distress syndrome. During the admission two months prior, bronchoscopy and bronchoalveolar lavage results showed evidence of acute and chronic granulomatous inflammation with necrotic debris but were negative for malignancy. Taken together, the possibility that this patient had an underlying chronic lung disease cannot necessarily be ruled out. The differential diagnoses include sarcoidosis, parenchymal lung disease, autoimmune interstitial lung disease such as systemic lupus erythematosus or vasculitis, or idiopathic pulmonary fibrosis related to the patient's smoking history. Results of human immunodeficiency virus serology, dihydrorhodamine testing for chronic granulomatous disease, and genetic screens for cystic fibrosis and alpha-1 antitrypsin were all negative; CD4 and CD8 flow cytometry and additional studies were planned, although the full workup was not completed due to the patient's sudden demise.

## Conclusions

Cepacia syndrome is a rare and lethal disorder, usually seen in susceptible cystic fibrosis and chronic granulomatous disease patient populations, but may occur in immunocompetent patients. The rapid decline and high mortality of cepacia syndrome present a challenge when managing these patients. Many individual factors complicated this case, including possible underlying conditions, a history of smoking, and the chronic nature of the infection. At the time of presentation, the patient's condition had already progressed to severe sepsis. These factors, coupled with the lethality of cepacia syndrome, led to an unfortunate outcome in this case despite appropriate initial management and vigorous resuscitative efforts. Unfortunately, this case was one of few published detailing an immunocompetent patient with cepacia syndrome. Although some data has been published regarding antibiotic effectiveness in Burkholderia organisms, most publications concern *B. pseudomallei*, which is known to cause infection in general patient populations more frequently. Studies for antibiotic optimization in immunocompetent *B. cepacia* patients would be ideal but are likely impractical due to the rarity and mortality of cases. *B. cepacia* is a rare but devastating pathogen, and its deadly potential must not be overlooked as an unlikely occurrence.
